# Cognitive-Motor Interference in Neurodegenerative Disease: A Narrative Review and Implications for Clinical Management

**DOI:** 10.3389/fpsyg.2018.02061

**Published:** 2018-10-29

**Authors:** Tara L. McIsaac, Nora E. Fritz, Lori Quinn, Lisa M. Muratori

**Affiliations:** ^1^Department of Physical Therapy, Arizona School of Health Sciences, A.T. Still University, Mesa, AZ, United States; ^2^Program in Physical Therapy and Department of Neurology, Wayne State University, Detroit, MI, United States; ^3^Department of Biobehavioral Sciences, Teachers College, Columbia University, New York, NY, United States; ^4^Department of Physical Therapy, SHTM, Stony Brook University, Stony Brook, NY, United States

**Keywords:** multitasking, Parkinson’s disease, multiple sclerosis, Huntington’s disease, attention

## Abstract

This paper provides a narrative review of cognitive motor interference in neurodegeneration, including brain imaging findings specific to interference effects in neurodegenerative disease, and dual task assessment and intervention in Parkinson’s disease (PD), multiple sclerosis (MS), and Huntington’s disease (HD). In a healthy central nervous system the ability to process information is limited. Limitations in capacity to select and attend to inputs influence the ability to prepare and perform multiple tasks. As a result, the system balances demands, switching attention to the most task-relevant information as it becomes available. Limitations may become more apparent in persons with neurodegenerative diseases (ND) with system-specific impairments in PD, MS, and HD. These ND affect both cognitive and motor function and are thus particularly susceptible to dual task interference. Issues related to performer and task characteristics and implications of these findings for both the standard assessment of dual task abilities as well as development and evaluation of interventions aimed at improving dual task ability are discussed. In addition, we address the need for optimizing individualized assessment, intervention and evaluation of dual task function by choosing cognitive and motor tasks and measures that are sensitive to and appropriate for the individual’s level of function. Finally, we use current evidence to outline a 5-step process of clinical decision making that uses the dual task taxonomy as a framework for assessment and intervention.

## Introduction

Every day people perform multiple tasks concurrently. Activities like walking and driving while engaged in a discussion require attention to several, sometimes competing actions with shifts and distribution of attention to control movements safely. The ability of the central nervous system to process this information is limited (Marois and Ivanoff, 2005) and influences the nervous system’s ability to prepare and perform tasks simultaneously. As a result, the system balances demands, switching attention to the most task-relevant information as it becomes available. Although present in healthy individuals, limitations become more apparent in persons with neurodegenerative disease with system-specific impairments noted in Parkinson’s disease (PD) (Dujardin et al., 2013), multiple sclerosis (MS) (Beatty et al., 1995), and Huntington’s disease (HD) (Thompson et al., 2010). These ND affect both cognitive and motor function and are thus particularly susceptible to dual task interference. Furthermore, as ND typically are diagnosed in mid- to late-life, the incidence is expected to soar as the population ages and will likely present greater demand for clinical management (Reitz et al., 2011; Reeve et al., 2014).

There is increasing focus on diagnostic approaches, and subsequent intervention development and selection, which are based on addressing motor impairments and resulting activity limitations without compartmentalizing patients primarily on medical diagnoses. For example, the National Institute of Mental Health (NIMH) is defining a new nosology that is based not solely on biology, but also key symptoms across levels of function. Similarly, the American Physical Therapy Association (APTA) is developing diagnoses based on movement system impairments that cut across common medical diagnoses. Indeed, despite etiological differences, many of the neuropathological changes seen in disease such as PD, MS, and HD affect similar processes, including the capacity available for attention to multiple tasks and directly or indirectly executive functioning.

The purpose of this paper is to examine cognitive-motor interference in neurodegenerative diseases (ND) and to discuss similarities across diseases with the aim of developing a common language for identifying dual task impairments. A narrative review of cognitive motor interference in neurodegeneration, including brain imaging findings specific to interference effects in neurodegenerative disease, and dual task assessment and intervention in PD, MS, and HD, serves as the foundation for a novel framework for clinical decision making in this population. Although studies typically focus on only a single ND rather than comparing differences and similarities among several ND, we suggest a need to explore similarities among ND and contrast CMI according to systems-related impairments. Issues related to performer and task characteristics and implications of these findings for both the standard assessment of dual task abilities as well as development and evaluation of interventions aimed at improving dual task ability are discussed.

## Neural Connectivity and Cognitive Motor Interference in ND

Although dual task deficits have been widely acknowledged in neurodegenerative disease, there is a paucity of knowledge of underlying dual task related connectivity changes. Much of what is known about dual task performance has been drawn from studies of elderly adults. Dual-task performance in the elderly has been associated with activation in the cerebellum (Wu et al., 2013; Blumen et al., 2014), precuneus (Wu et al., 2013; Blumen et al., 2014), superior parietal lobe (Burki et al., 2017), SMA (Blumen et al., 2014), and other prefrontal regions (Blumen et al., 2014; Al-Yahya et al., 2015). In particular, dual task walking has been specifically associated with greater functional connectivity in the SMA and prefrontal regions compared to single task walking in elderly adults on resting state fMRI (Yuan et al., 2015). However, others posit that no distinct brain areas are associated with dual-task performance; rather, performance depends on the interaction of the specific brain areas activated by the individual component tasks (Wantanabe and Funahashi, 2018).

The potential need for activation of multiple cortical areas to achieve optimal dual task performance suggests cortical neural degeneration may relate to specific dual task interference effects. In MS, for example, individuals are more frequently impaired on measures of sustained attention and visuospatial perception, and less frequently impaired on measures of language and immediate and remote memory (Rao et al., 1991). Attention impairment in people with relapsing-remitting MS is related to slowed central processing, including impairment of automatic and controlled processing of information, which may be present in all stages of disease (Balsimelli et al., 2007). Despite weak correlation with disease duration and physical disability status, the degree of cognitive impairment in MS has been related to the extent of topographically specific neuronal tissue damage and loss (Rogers and Panegyres, 2007).

Neuroimaging studies of dual task performance in individuals with PD have shown increased activity compared with controls in the cerebellum, PMC, parietal cortex, precuneus, and prefrontal areas (Wu and Hallett, 2008). Specific regions of the cerebellum, namely the vermis and lobule V, are likely involved with integration of networks associated with motor and cognitive functions. These regions seem to modify the integrated networks for improved efficiency with fewer neural demands to achieve the same performance during dual tasking in healthy participants (Wu et al., 2013; Gao et al., 2017; Leone et al., 2017). In contrast, individuals with PD have increased activity compared with controls in these cerebellar regions for single motor tasks but no additionally activated cerebellar regions during dual task performance (Gao et al., 2017). These findings in PD suggest that the limited cerebellar resources are consumed for single tasks and further activation for dual task performance is unavailable for integrating motor and cognitive networks. Dual task walking is particularly challenging for individuals with PD who experience freezing of gait (FOG) (Spildooren et al., 2010), suggesting that attentional control plays a key role in FOG. Imaging studies demonstrated reduced functional connectivity between the caudate and superior temporal lobe and hypo-connectivity between the dorsal putamen and precuneus that was related to worse dual-task performance (Vervoort et al., 2016). Furthermore, dual-task interference in individuals with FOG was correlated with lateralization of the pedunculopontine nucleus (PPN) structural connectivity (Peterson et al., 2015), supporting the suggestion that the PPN plays a role in attentional control (Yarnall et al., 2011).

In PD, attentional demands may exceed available resources in tasks that depend on internal cues (Brown and Marsden, 1988). Several of the hallmark deficits in PD are due to changes in the frontal-striatal circuits and involve executive defects in planning, initiation, and monitoring of goal-directed behaviors and working-memory. Visuospatial and memory deficits more representative of posterior cortical functioning are also present in persons with PD even without corresponding dementia (Pagonabarraga and Kulisevsky, 2012). In an experiment where motor and cognitive tasks were performed independently and combined in a dual task paradigm, individuals with PD showed distinct striatal recruitment that was not seen in single task performance or in the control participants. Results suggest that individuals with PD may have specific impairments of the cortical-striatal circuitry related to segregation needed to allow independence of motor and cognitive functions during dual tasking (Nieuwhof et al., 2017).

Given the challenge of performing dual tasks in the MRI scanner, fNIRS, EEG, and MEG have been employed to assess neural networks in real time during walking or standing dual tasks. These studies show increased activation in the prefrontal or frontal cortex with dual task walking (Holtzer et al., 2011; Mirelman et al., 2014). Of interest, older adults demonstrated different responses to dual tasks, showing substantial decreases in prefrontal activation compared to young adults (Holtzer et al., 2011; Beurskens et al., 2014). This finding was reproduced in persons with MS; individuals with MS demonstrate greater elevations in prefrontal cortex activation levels during dual-task walking compared to single-task walking than healthy controls (Hernandez et al., 2016). An fMRI study examining cognitive-cognitive dual-tasks in persons with MS found reduced prefrontal and parietal cortical activity that was associated with behavioral performance on tests of attention and executive function (Nebel et al., 2007).

Similar to MS and PD, HD results in motor and cognitive deficits during single task and dual task performance. Selective neuronal death in the cortex and striatum leads to progressive loss of function (Cowan and Raymond, 2006). Although general cognitive changes are seen across the spectrum of HD and may be an early sign of disease (Carlozzi et al., 2011) studies show speed of processing, initiation, and measures of attention may be better able to capture the onset of functional decline in HD (Peavy et al., 2010). Specific impairments in self-generated maintenance of attention may be especially important in the assessment and treatment of multitasking in HD. Problems with simultaneous monitoring of multiple input channels in a divided attention task, set-shifting deficits and the inability to use multimodal information (Sprengelmeyer et al., 1995) suggests that attentional disturbances may be a primary cause of dual task conflicts in HD. Indeed, individuals with HD demonstrate a switching deficit even when the switch is predictable and not time-constrained, indicating a switching deficit distinct from PD and possibly related to executive control default to “response set” (Aron et al., 2003). Imaging studies of neural networks underlying dual task performance are lacking in individuals with HD. One study explored the effect of dual-task walking on EEG parameters in persons with HD and found an increase in the P3 amplitude with walking that was inversely correlated with motor impairment (de Tommaso et al., 2017). These findings suggest that cortical activation was facilitated in a combined motor-cognitive dual task but decreased as motor impairment increased in participants with HD.

To accomplish challenging tasks, including dual tasks, neural networks must flexibly adapt to the demands of the task. Several models of attentional and executive function networks needed for dual task performance have been proposed (Posner and Petersen, 1990; Miyake et al., 2000; McDowd, 2007). The flexible shifting, switching, or division of attention between tasks and the inhibition of information when appropriate leads to successful dual-task performance. The executive control network responsible for allocating attention to task demands has been associated with the prefrontal cortex, anterior cingulate gyrus, other frontal areas (Posner et al., 2006) and parietal areas (Petersen and Posner, 2012), which aligns with neuroimaging work exploring dual tasks in healthy adults.

In the setting of neurodegeneration, there is a loss of neurons within these attention and executive function networks, leading to an overall reduction in the plasticity of the network. A loss in the flexibility of the network to adapt to the demands of challenging dual tasks may explain the impairments seen across ND. There is some evidence of this among individuals with PD, who demonstrate reduced efficiency in neural coding (Wu et al., 2015) as well as greater activation (i.e., greater recruitment of resources) than controls, even when performing automatic tasks (Wu and Hallett, 2005). Reaching a resource ceiling has particular clinical implications for individuals with neurodegenerative disease as they progress through their disease course.

## Clinical Considerations of Dual Task Performance in ND

### Tests and Measures

The effect of performing two tasks simultaneously compared with performance of each task alone is measured as a dual task effect (DTE). Such measurement reveals a cost or benefit to task performance and is an indication of interference or facilitation, respectively, of the limited capacity for attention and information processing. The DTE is a relative measure of an outcome of interest (e.g., gait speed) for each task, with a positive multiplier for variables for which higher values indicate improved performance and a negative multiplier for variables for which higher values indicate worse performance (Kelly et al., 2010). The DTE can be visualized using performance operating characteristic plots that represent the interaction of two tasks and to what degree each task is prioritized relative to the other, a between task trade-off (Kelly et al., 2010).

When assessing dual task function and CMI it is important to choose measures and tasks that are sensitive to specific impairments for that individual. While the inclination for clinicians has been to recognize general categories of impairments according to disease (e.g., bradykinesia and set-switching/attention in PD, dyscoordination and slowed processing speed in MS, hyperkinesia and working memory in HD); the notable heterogeneity of all three diseases and the impairments that are common among them lend support to using systems impairment categories for determining clinical measures and assessment rather than diagnostic criteria. For example, using a serial-7 subtraction task while performing the timed up and go (TUG) task reveals an individual’s capability for working memory and attention while walking (Bristow et al., 2016). Alternatively, the Stroop task indicates the ability to selectively inhibit automatic responses in favor of goal-directed ones during functional mobility. Importantly, selection of the appropriate version of the Stroop, visual or auditory, is key to assessing the specific modality impairment of the performer, regardless of medical diagnosis. Performing the walking Trail Making Test (TMT) (Perrochon and Kemoun, 2014) can highlight difficulties in speed of processing (TMT-A) and with mental flexibility and complex attention (TMT-B). Verbal fluency capability can be assessed by timed naming of items (e.g., animals, plants, and foods). Such cognitive tests are influenced by the individual’s impairments and by their inherent capabilities and experiences, possibly more than their medical diagnosis.

Measurement of seated dual task activities such as driving, when concerns of balance and gait are eliminated, are primarily limited to driving simulator programs (Campos et al., 2017). While these programs offer assessments of multisensory, multidimensional, and complex task performance in a simulated “real-world” environment, they do not specifically focus on mechanisms of CMI, and are difficult to directly compare results with studies of dual task paradigms in neurorehabilitation. A recently developed measurement for seated dual-task activity, developed for use in people with HD and being tested in PD, is the Moneybox Test (MBT). Subjects transfer coins in order of size, value, and with and without concurrently reciting the alphabet (Clinch et al., 2018). The MBT was shown to be sensitive in early stage HD and may prove useful in identifying CMI when seated using primarily the upper extremities and without the requirement to control standing balance or walking among ND.

Little has been reported on how specific cognitive domains interact with aspects of movement in dual task behaviors, particularly for individuals with ND. However, in a recent study exploring associations between several cognitive domains and gait variability in people with MS, Kalron et al. (2018) found that global cognition, executive function subcategory, and cognitive motor skills were associated with step time variability in non-fallers with MS, but no associations for the fallers. Exploring similar associations in people with PD, Stegemöller et al. (2014) found that cognitive processing speed correlated with stride length and walking speed, and executive function correlated with step width variability. Working memory was not associated with any gait measures. In studies of people with HD, Kloos et al. (2017) found an association between executive function (Stroop Interference and Symbol Digit Modalities Test) and the Tinetti Mobility Test of balance and gait function. Thus, evidence is emerging for a non-diagnosis specific relationship between cognitive and motor functions (e.g., executive function with step variability in MS and PD) and suggesting assessment of dual task function and CMI according to systems impairments may be more relevant clinically.

Among the currently recommended dual task outcome measures is the TUG-Cognitive, a “highly recommended measure” from both the MS and the PD Evidence Database to Guide Effectiveness (MS-EDGE, 2012; PD-EDGE, 2014) of the APTA. Although not measures of dual task *per se*, the Stroop, Symbol Digit Modalities Test, Category Verbal Fluency Test, and the TMT have been recommended and optimized for assessing cognitive function in HD, and the TUG, Tinetti Mobility Test, Four Square Step Test, Berg Balance Scale, and Physical Performance Test for assessing mobility in people with HD (Quinn et al., 2013; Kloos et al., 2014; Stout et al., 2014). Other tests of dual task function that are used clinically, but not specifically recommended for individuals with neurodegenerative disorders, include the Walking and Remembering Test, Stops Walking When Talking test, and the Walking While Talking Test (Beauchet et al., 2009; McCulloch et al., 2009; Fritz et al., 2016).

While dual task training and outcomes related to dual task ability is receiving increasing attention in the literature, significant gaps remain that limit our ability to make concrete clinical recommendations. Importantly, virtually all studies have involved gait and/or balance tasks, and there is a paucity of information pertaining to dual task ability involving upper extremity movements as evaluated by the MBT (Clinch et al., 2018). Ecologically valid outcome measures that reflect dual task abilities are lacking. Gaps in measurement for dual task performance include the lack of longitudinal assessments; lack of exploring relationships of systems impairments, rather than medical diagnoses, and dual task performance; lack of assessments across different motor tasks and across multiple cognitive domains; and few formal assessments to examine the influence of input and output modality on performance. Preliminary studies are underway to ameliorate several of these gaps; long-term screening and assessment of individuals with MS, PD, and HD on a battery of motor, cognitive and dual task measures have been initiated; motor-cognitive dual tasks are being examined in standing, walking, and across multiple cognitive domains; and prospective falls data is being collected by one author to identify relationships among dual-task performance and risk of future falls.

### Dual Task Interventions for Neurodegenerative Disease

Over the past 10 years there has been increasing attention to studies that have evaluated interventions to improve dual task ability in individuals with ND. Killane et al. (2015) evaluated community dwelling participants with PD to examine the effect of dual motor-cognitive virtual reality training on dual task performance. Participants completed eight 20-min intervention sessions consisting of a virtual reality maze while performing a cognitive task. A significant improvement was found in dual task cognitive and motor performance, but only for those individuals with PD who experienced freezing of gait. In a systematic review of 21 studies evaluating dual task intervention in individuals with ND, preliminary data supports the use of dual task training for individuals with MS, PD, AD, and dementia (Wajda et al., 2017). The authors categorized dual task interventions into three types: (1) multi-modal exercise interventions, with the underlying tenet that dual task performance could be explicitly improved following direct practice of divided attention; (2) virtual reality and exergaming training, in which participants were immersed in virtual environments which allowed them to encounter objects or characteristics that required attention; and (3) cueing training, in which participants were either provided verbal cues (e.g., to take bigger steps), or were provided with music while walking. A range of different training modalities appear to be beneficial, ranging from simply adding a cognitive task while walking to utilizing virtual reality environments to simulate complex, real-life scenarios that patients may encounter in their day-to-day life.

Despite the positive conclusions above, the recent Duality trial suggests that there is no benefit of dual task training over single task training in people with PD (Strouwen et al., 2017). Significant improvements were reported in dual task gait velocity in both consecutive training and dual task training groups, and the authors suggest that either consecutive or integrated dual task training can be beneficial without increasing fall risk. Similarly, evidence is lacking in support of single versus dual task training in individuals with MS. Monjezi et al. (2017) reported no benefit of dual task training over single task training for balance training with and without a cognitive task. Sosnoff et al. (2017) also reported no clear benefit of dual task versus single task training on gait and balance tasks in a randomized feasibility study in people with MS, although there was a trend for better performance by participants in the dual task training group.

There is some preliminary evidence in support of dual task training’s effect on outcomes other than dual task abilities, such as falls. Yitayeh and Teshome (2016) conducted a systematic review to determine the effectiveness of physical therapy interventions to address balance impairment and postural instability in persons with idiopathic PD. The authors reported that training that incorporated both dual tasking and PD-specific balance components significantly benefited balance and gait abilities when compared with usual care. In addition, dual task activities resulted in both a decreased fall rate and fear of falling. Fritz et al. (2015) further reported that dual task training has a range of benefits in individuals with ND. Dual task training was found to improve single task gait velocity and stride length in subjects with PD and AD, and may improve balance and cognition in those with PD and AD. Highly challenging balance training that incorporates dual task training has been shown to be beneficial for individuals with mild to moderate PD compared to usual care (Conradsson et al., 2015). While Fritz et al. (2016) has demonstrated that dual task assessment can be useful in identifying fall risk in HD, there are no papers specifically addressing dual task training in HD.

The presence of a dual task impairment does not immediately suggest that it is amenable to change. Several factors must be considered to facilitate decision-making; these include:

(1) Environmental considerations. If an individual spends a considerable amount of their time in situations where the environment is relatively consistent and non-variable, and their routine has little variability day to day, then the type and degree of dual task training is likely to be different compared to individuals who encounter more day to day variability in their environmental conditions.
(2) Task considerations. Dual tasking may be more important in certain tasks than others. For example, falls risk is known to be increased while performing transitional tasks (e.g., sit to stand) or during certain environment conditions (e.g., low lighting). Therefore, training on dual tasks should be based on a risk assessment and should incorporate activities likely to be encountered.
(3) Performer considerations. The degree of dual task impairment may impact on an individual’s ability to benefit from dual task training. If the impairment in either dual tasking or performing either task individually is over a certain threshold it may be best to consider compensatory strategies (e g. avoiding dual task situations altogether). For example, individuals who have significant impairments in cognition (e.g., MMSE below 21) may benefit more from developing compensatory strategies than the time and effort needed to train dual tasks. Furthermore, typical neurodegenerative disease progression involves impairments in learning and re-learning skills. Toward the middle and later stages, when there is typically wide spread cortical and subcortical damage in most ND, learning may be sufficiently impaired to prevent or significantly limit an individual’s ability to learn strategies to divide attention in a safe and effective manner.

### A Novel Framework for Examining Cognitive Motor Interference in ND

We have previously presented a dual task taxonomy based on a definition of dual tasking as the concurrent performance of two tasks that can be performed independently and have distinct and separate goals (McIsaac et al., 2015). Individual tasks are separated into simple and complex and consideration is given to the degree of task novelty to the performer. Indeed, a highly familiar pairing of two simple tasks, like brushing teeth while watching the news, may be easier for someone to perform than a highly complex single task, like walking across an ice rink. Likewise, “simple” combinations of tasks for one individual, like walking across a crowded street while engaged in a cell-phone conversation, may show little or no interference effects, while the same dual task activity for a person with neurodegenerative disease might be quite impaired. The amount of interference one task has on another scales with the complexity and novelty of the tasks involved. For example, Langhanns and Müller (2017) demonstrated that an instruction to “stay stock-still” during a calculation task required more cortical processing than performing the same cognitive task while sitting or lying down at rest. The explicit instruction to restrict all movement, although not motorically complex, increased the novelty of the sitting task and, therefore, required more cognitive effort for the participants.

**FIGURE 1:**
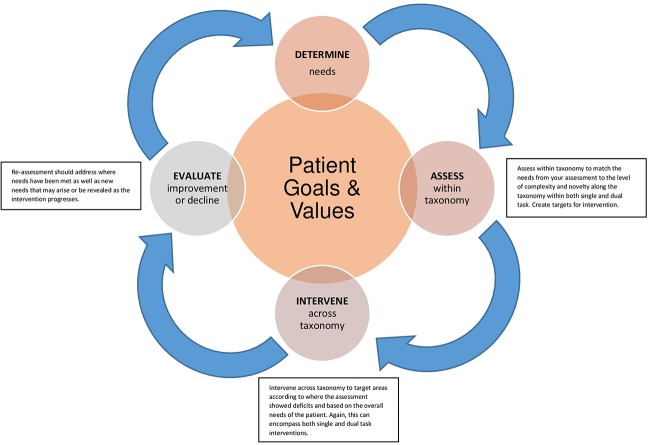
Legend – Framework for assessment and intervention with dual task. The diagram begins in the center with (1) patient goals and values and projects to the clinician’s role to (2) determine needs. At point (3) the clinician can match the needs from an assessment to the level of complexity and novelty along the dual task taxonomy to create targets for (4) intervention. (5) Re-assessment should address where needs have been met as well as new needs that may arise or be revealed as the intervention progresses.

For individuals with ND, determining when and how to address dual task impairments should be implemented is a multistep process that starts with the individual and is continually assessed during the disease process. An important factor when considering dual task assessment and intervention is to determine if restorative strategies to improve dual task function are warranted. In some cases, use of compensatory strategies, such as avoidance of complex dual task situations, may be recommended. Figure 1 provides a schematic of this process using the dual task taxonomy as a framework for assessment and intervention.

Each step of the process takes the status of the individual into consideration, beginning in Step 1 with understanding the patient’s values and goals for treatment. From that foundation, clinicians can determine the needs (Step 2) and assess how the patient performs along different levels of the taxonomy (Step 3), noting effects of novelty and complexity on performance. Personalized interventions can be implemented in Step 4 to match the patient goals and target the specific gaps in performance. Importantly, the process continues to re-assessment (Step 5) to emphasize the continued need to monitor and measure changes in performance in neurodegenerative disease as there is rarely a linear benefit from intervention.

The patterns of performance deterioration when a cognitive task and a motor task are simultaneously performed (cognitive-motor interference; CMI) has been previously described (Plummer et al., 2013). Dual task performance may encompass any combination of motor and cognitive tasks, i.e., motor-motor, motor-cognitive, or cognitive-cognitive. In addition to spatial and object-specific considerations, DTEs depend on the pairings of stimuli and response modalities (e.g., visual stimuli requiring tactile response) (Stelzel and Schubert, 2011). These effects may be further defined by dependence (or interdependence) in working memory. As shown in early descriptions of a working memory system, tasks with similar resource demands may create conflicts within separate and/or competing working memory domains with limited resources (see Baddeley, 1986). Hazeltine and Wifall (2011) showed this interaction between sensory modality and working memory in a study demonstrating that vocal responses interfered with working memory for sound while manual responses interfered with working memory for location. Therefore, modality pairings may contribute to dual-task performance by creating further competition for available resources regardless of whether capacity is from single or multiple resources.

The framework highlights the importance of examining components of dual task performance at the level of performer capabilities and task requirements so that areas of potential resource overlap and interference can be appreciated. The framework also provides a common language with which to measure, assess, and deliver interventions for CMI and dual task performance from the perspective of systems-related impairments rather than diagnosis, in line with the development of NIMH’s new nosology and the APTA’s movement systems diagnoses.

## Conclusion

The ability to prepare and perform multiple tasks in day to day activity requires the capacity to select, attend to and process information related to the goals for the activity. Limitations in this capacity lead to dual task interference and reduced task performance. The increased difficulty in dual task function for people with ND may be related to system-specific impairments and the pathologic changes in underlying neural networks. The type of tasks paired (motor, cognitive, or both); the specific spatial and object-specific characteristics of the tasks; the postural and gait configurations when carrying out the tasks (seated, standing, and walking); the pairing of modalities used to perceive stimuli and with which to respond (e.g., vision, hearing, and touch) are all considerations in assessing dual task function and developing optimal clinical interventions and rehabilitation strategies. We have reviewed system-specific aspects of motor and cognitive function in MS, PD, and HD and the related underlying neural networks, and summarized possible effects on cognitive motor interference. Optimizing individualized assessment, intervention and evaluation of dual task function requires choosing cognitive and motor tasks and measures that are sensitive to and appropriate for the individual’s level of disease and modality involvement. We discuss some measurement tools commonly used for motor, cognitive and dual tasks, but more studies on the psychometric properties of measures of dual task function in ND are needed. The current preliminary evidence from a small number of studies in MS, PD, and HD support the beneficial influence of dual task training, but variability among training methods and lack of standardized incorporation of cognitive tasks into the training protocols leaves limited ability to interpret overall findings. We suggest future research include assessment of similarities among ND and comparisons according to systems impairments instead of medical diagnoses, rather than focusing on a single ND. This has begun to occur in systematic reviews (e.g., Wajda et al., 2017), and we encourage such cross-diagnosis assessments of commonalities in areas from neural networks to dual task performance and intervention. Lastly, we outline a 5-step process of clinical decision making that uses the dual task taxonomy as a framework for assessment and intervention and takes into account the environmental, task, and performer considerations.

## Author Contributions

TM, NF, LQ, and LM contributed to the conceptualization and writing of this manuscript and agreed to be accountable for all aspects of the work.

## Conflict of Interest Statement

The authors declare that the research was conducted in the absence of any commercial or financial relationships that could be construed as a potential conflict of interest.
